# Supplementary material for: Evaluation of therapeutic effect of oral Ursodeoxycholic Acid on indirect hyperbilirubinemia in term neonates undergoing phototherapy: A randomized controlled clinical trial
Source: PLoS One. 2023 Dec 12;18(12):e0273516. doi: 10.1371/journal.pone.0273516 (PMC10715657; doi:10.1371/journal.pone.0273516)
Supplement: S1 File — (PDF) [file pone.0273516.s003.pdf]

پروتکل کارآزمایی بالینی
مرکز ثبت کارآزمایی بالینی ایران

۱۴۰۱/۰۱/۰۷

بررسی اثر درمانی داروی اورسودئوکسی کولیک اسید خوراکی بر هیپربیلی روبینمی غیرمستقیم در نوزادان ترم تحت درمان فوتو تراپی بستری در بیمارستان 17شهریور - یک مطالعه کارآزمایی بالینی شاهد دار تصادفی شده

چکیده پروتکل

هدف ازمطالعه

مقایسه داروی اورسو خوراکی به همراه فو توتراپی با فوتوتراپی تنها در کاهش هیپربیلی روبینمی غیرمستقیم در نوزادان ترم تحت درمان فوتو تراپی بستری در بیمارستان 17شهریور

طراحی

کارآزمایی بالینی دارای گروه کنترل، بدون کورسازی، تصادفی شده، فاز ۳، بر روی ۱۰۶ بیمار. برای تصادفی سازی از نرم افزارsealedenvelope استفاده می شود.

نحوه و محل انجام مطالعه

این تحقیق يك مطالعه كارآزمايي باليني تصادفی شده است که پس از اخذ کد اخلاق به مدت 8 ماه در بیمارستان 17 شهریور شهر رشت روی نوزادان ترم 3 تا 7 روزه که بدلیل زردی در بخش نوزادان بستری می شوند انجام خواهد شد. پس از انتخاب نوزادان با توجه به معیارهای ورود و خروج، رضایت نامه کتبی از والدین گرفته خواهد شد. بیماران در 2 گروه تقسیم بندی خواهند شد. گروه A دریافت کننده داروی اورسو خوراکی به همراه فوتو تراپی و گروهB دریافت کننده فوتوتراپی به تنهایی (کنترل) خواهند بود. مصرف دارو توسط رزیدنت کودکان ارزیابی و ویزیت انجام شده و درصورت بروز عوارض، دارو قطع می شود. 1-اورسودئوکسی کولیک اسید خوراکی با دوز 10 ميلي گرم به ازاي هر كيلوگرم بصورت منقسم هر 12 ساعت و در زمان بستری تجویز می شود که این دوز در شیر مادر حل می شود.

شرکت کنندگان/شرایط ورود و عدم ورود

معیار ورود شامل: رضایت کامل والدین از حضورکودکان در مطالعه،وزن زمان تولد:2500 تا 4000 گرم،تغذیه ی انحصاری با شیر مادر،سن بارداری 38 تا 41 هفته،سن 3 تا 7 روز،بیلی روبین توتال 14 تا 20 و مستقیم کمتر از 2 می باشد. معیارهای عدم ورود شامل: ناسازگاری ABOو RH،کمبود آنزیم G6PD،هیپربیلی روبینمی مستقیم،سپتیسمی.

گروه‌های مداخله

داروی اورسو با دوز ۱۰ میلی گرم بر کیلوگرم روزانه در نوزادانی که تحت فوتو تراپی قرار میگیرند در طی مدت بستری

متغیرهای پیامد اصلی

پیامد اولیه میزان بیلی روبین بوده که در طی سه تا چهار روز بستری ثبت خواهد شد. پیامد ثانویه مدت زمان بستری- عوارض دارویی و مقاطع زمانی سنجش بیلی روبین است.

اطلاعات عمومی

علت بروز رسانی

نام اختصاری

اطلاعات ثبت در مرکز

شماره ثبت کارآزمایی در مرکز: **IRCT20210201050199N1**

تاریخ تایید ثبت در مرکز: **2021-04-03, ۱۴۰۰/۰۱/۱۴**

زمان‌بندی ثبت: **prospective**

آخرین بروز رسانی: **2021-04-03, ۱۴۰۰/۰۱/۱۴**

تعداد بروز رسانی‌ها: **0**

تاریخ تایید ثبت در مرکز

2021-04-03, ۱۴۰۰/۰۱/۱۴

اطلاعات تماس ثبت کننده

نام

منیژه تبریزی

نام سازمان / نهاد

کشور

جمهوری اسلامی ایران

تلفن

+98 13 3336 9002

آدرس ایمیل

drs.tabrizi@gmail.com

وضعیت بیمار گیری

بیمار گیری تمام شده

منبع مالی

تاریخ شروع بیمار گیری مورد انتظار

2021-04-19, ۱۴۰۰/۰۱/۳۰

تاریخ پایان بیمار گیری مورد انتظار

2021-10-22, ۱۴۰۰/۰۷/۳۰

تاریخ شروع بیمارگیری تحقق یافته

خالی

تاریخ پایان بیمارگیری تحقق یافته

خالی

تاریخ خاتمه کارآزمایی

خالی

عنوان علمی کارآزمایی

بررسی اثر درمانی داروی اورسودئوکسی کولیک اسید خوراکی بر هیپربیلی روبینمی غیرمستقیم در نوزادان ترم تحت درمان فوتو تراپی بستری در بیمارستان 17شهریور -یک مطالعه کارآزمایی بالینی شاهد دار تصادفی شده

عنوان عمومی کارآزمایی

بررسی اثر درمانی داروی اورسودئوکسی کولیک اسید خوراکی بر هیپربیلی روبینمی غیرمستقیم در نوزادان ترم تحت درمان فوتو تراپی بستری در بیمارستان 17شهریور -یک مطالعه کارآزمایی بالینی شاهد دار تصادفی شده

هدف اصلی مطالعه

درمانی

شرایط عمده ورود و عدم ورود به مطالعه

شرایط عمده ورود به مطالعه قبل از تصادفی سازی:

رضایت کامل والدین از حضورکودکان در مطالعه وزن زمان تولد 2500 تا 4000 گرم تغذیه ی انحصاری با شیر مادر سن بارداری 38 تا 41 هفته سن 3 تا 7 روز بیلی روبین توتال 14 تا 20 و مستقیم کمتر از 2

شرایط عمده عدم ورود به مطالعه قبل از تصادفی سازی:

ناسازگاری ABOو RH کمبود انزیم گلوکوز 6 فسفات دهیدروژناز هیپربیلی روبینمی مستقیم سپتیسمی بیماری هایی که منجر به هیپربیلی روبینمی می شوند (سندرم کریگلر نجار،سندرم ژیلبرت، هیپرتیروئیدی، هیپوتیروئیدی، بیماریهای کبدی) پره ترم ها نوزاد مادر دیابتی

سن

از سن **3 روزه** تا سن **7 روزه**

جنسیت

هر دو

فاز مطالعه

3

گروه‌های کور شده در مطالعه

اطلاعات موجود نیست

حجم نمونه کل

حجم نمونه پیش‌بینی شده: **106**

تصادفی سازی (نظر محقق)

اختصاص تصادفی به گروه‌های مداخله و کنترل

توصیف نحوه تصادفی سازی

تصادفی سازی قبل از شروع برنامه به وسیله نرم افزار https://www.sealedenvelope.com با در نظر گرفتن 53 نفر در هر 2گروه (گروه اورسو و فوتو و کنترل) در بلوک های 4 تایی انجام خواهد شد و هر یک از آنها داخل پاکتی جداگانه طبق لیست حاصل از نرم افزار قرار گرفته و در آن بسته می شود و در اختیارشخص سوم قرار خواهد گرفت .در صورت مراجعه بیمار و واجد شرایط بودن او، پاکت باز شده وبراساس توالی موردنظر بدون اطلاع از درمان بعدی مورد درمان قرار می گیرد. لازم به ذکر است با توجه به گروه بندی انجام شده بیماران بر اساس تخصیص تصادفی که با نرم افزار انجام شده است در 2 گروه تقسیم بندی خواهند شد. بررسی سایر متغیر ها و متغیرهای وابسته به هیپربیلی روبینمی و برسی عوارض دارو بصورت روزانه در طول بستری بر عهده رزیدنت کودکان می باشدواجرای دستورات وداروها با پرستار بخش می باشد. گروه A دریافت کننده داروی اورسو خوراکی به همراه فوتو تراپی و گروهB دریافت کننده فوتوتراپی به تنهایی (کنترل) خواهند بود. • 1, 4, 1, Group B • 1, 4, 2, Group B • 1, 4, 3, Group A • 1, 4, 4, Group A • 2, 4, 1, Group B

کور سازی (به نظر محقق)

کور نشده است

توصیف نحوه کور سازی

دارو نما

ندارد

اختصاص به گروه‌های مطالعه

موازی

سایر مشخصات طراحی مطالعه

کد ثبت در سایر مراکز ثبت بین‌المللی

خالی

تاییدیه کمیته‌های اخلاق

1

کمیته اخلاق

نام کمیته اخلاق

کمیته اخلاق معاونت پژوهشی دانشگاه علوم پزشکی گیلان

آدرس خیابان

سیادتی

شهر

رشت

استان

گیلان

کد پستی

4144444444

تاریخ تایید

2021-03-03, ۱۳۹۹/۱۲/۱۳

کد کمیته اخلاق

IR.GUMS.REC.1399.645

بیماری‌های (موضوعات) مورد مطالعه

1

شرح

هیپر بیلی روبینمی

کد ICD-10

E80.6

توصیف کد ICD-10

Other disorders of bilirubin metabolism

متغیر پیامد اولیه

1

شرح متغیر پیامد

بیلی روبین توتال

مقاطع زمانی اندازه‌گیری

هر 24 ساعت

نحوه اندازه‌گیری متغیر

دستگاه اوتوآنالایزر

متغیر پیامد ثانویه

1

شرح متغیر پیامد

بیلی روبین غیرمستقیم

مقاطع زمانی اندازه‌گیری

هر 24 ساعت

نحوه اندازه‌گیری متغیر

دستگاه اتو انالایزر

گروه‌های مداخله

1

شرح مداخله

گروه مداخله: دریافت کننده داروی اورسو خوراکی به همراه فوتو تراپی (تجویز اورسودئوکسی کولیک اسید خوراکی با دوز 10 میلي گرم به ازاي هر کیلوگرم بصورت منقسم هر 12 ساعت و در زمان بستری بصورت حل شده در شیر مادر)

طبقه بندی

درمانی - داروها

2

شرح مداخله

گروه کنترل: دریافت کننده فوتوتراپی به تنهایی

طبقه بندی

درمانی - داروها

مراکز بیمار گیری

1

مرکز بیمار گیری

نام مرکز بیمار گیری

بیمارستان کودکان 17 شهریور

نام کامل فرد مسوول

منیژه تبریزی

آدرس خیابان

سیادتی

شهر

رشت

استان

گیلان

کد پستی

4144444444

تلفن

+98 13 3336 9002

ایمیل

drs.tabrizi@gmail.com

حمایت کنندگان / منابع مالی

1

حمایت کننده مالی

نام سازمان / نهاد

دانشگاه علوم پزشکی رشت

نام کامل فرد مسوول

محمدرضا نقی پور

آدرس خیابان

سیادتی

شهر

رشت

استان

گیلان

کد پستی

4144444444

تلفن

+98 13 3336 9002

ایمیل

research@gums.ac.ir

ردیف بودجه

کد بودجه

آیا منبع مالی همان سازمان یا نهاد حمایت کننده مالی است؟

بلی

عنوان منبع مالی

دانشگاه علوم پزشکی رشت

درصد تامین مالی مطالعه توسط این منبع

100

بخش عمومی یا خصوصی

عمومی

مبدا اعتبار از داخل یا خارج کشور

داخلی

طبقه بندی منابع اعتبار خارجی

خالی

کشور مبدا

طبقه بندی موسسه تامین کننده اعتبار

دانشگاهی

فرد مسوول پاسخگویی عمومی کارآزمایی

اطلاعات تماس

نام سازمان / نهاد

دانشگاه علوم پزشکی رشت

نام کامل فرد مسوول

منیژه تبریزی

موقعیت شغلی

استادیار

آخرین مدرک تحصیلی

متخصص

سایر حوزه‌های کاری/تخصص‌ها

کودکان

آدرس خیابان

سیادتی

شهر

رشت

استان

گیلان

کد پستی

4144444444

تلفن

+98 13 3336 9002

فکس

ایمیل

drs.tabrizi@gmail.com

فرد مسوول پاسخگویی علمی مطالعه

اطلاعات تماس

نام سازمان / نهاد

دانشگاه علوم پزشکی رشت

نام کامل فرد مسوول

منیژه تبریزی

موقعیت شغلی

استادیار

آخرین مدرک تحصیلی

متخصص

سایر حوزه‌های کاری/تخصص‌ها

کودکان

آدرس خیابان

سیادتی

شهر

رشت

استان

گیلان

کد پستی
4144444444
تلفن
+98 13 3336 9002
فکس
ایمیل
drs.tabrizi@gmail.com

فرد مسوول به‌روز رسانی اطلاعات

اطلاعات تماس
نام سازمان / نهاد
دانشگاه علوم پزشکی رشت
نام کامل فرد مسوول
منیژه تبریزی
موقعیت شغلی
استادیار
آخرین مدرک تحصیلی
متخصص
سایر حوزه‌های کاری/تخصص‌ها
کودکان
آدرس خیابان
سیادتی
شهر
رشت
استان
گیلان
کد پستی
4144444444
تلفن
+98 13 3336 9002
فکس
ایمیل
drs.tabrizi@gmail.com

برنامه انتشار

فایل داده شرکت کنندگان (IPD)
خیر - برنامه‌ای برای انتشار آن وجود ندارد
توجیه/علت عدم تصمیم/عدم انتشار IPD
به دلیل مسایل اخلاقی ومحرمانه بودن اطلاعات
پروتکل مطالعه
خیر - برنامه‌ای برای انتشار آن وجود ندارد
نقشه آنالیز آماری
خیر - برنامه‌ای برای انتشار آن وجود ندارد
فرم رضایتنامه آگاهانه
خیر - برنامه‌ای برای انتشار آن وجود ندارد
گزارش مطالعه بالینی
خیر - برنامه‌ای برای انتشار آن وجود ندارد
کدهای استفاده شده در آنالیز
خیر - برنامه‌ای برای انتشار آن وجود ندارد
نظام دسته‌بندی داده (دیکشنری داده)
خیر - برنامه‌ای برای انتشار آن وجود ندارد
