# Supplementary material for: Evaluation of therapeutic effect of oral Ursodeoxycholic Acid on indirect hyperbilirubinemia in term neonates undergoing phototherapy: A randomized controlled clinical trial
Source: PLoS One. 2023 Dec 12;18(12):e0273516. doi: 10.1371/journal.pone.0273516 (PMC10715657; doi:10.1371/journal.pone.0273516)
Supplement: S2 File — (PDF) [file pone.0273516.s004.pdf]

Clinical Trial Protocol
Iranian Registry of Clinical Trials

27 Mar 2022

Therapeutic effect of oral ursodeoxycholic acid on indirect hyperbilirubinemia in full-term neonates undergoing phototherapy in hospital 17 Shahrivar - a randomized controlled clinical trial

Protocol summary

Study aim

Comparison of oral Urso with phototherapy and phototherapy alone in indirect hyperbilirubinemia reduction in term neonates undergoing phototherapy in hospital 17 Shahrivar

Design

A clinical trial with the control group, without blinding, randomized, phase 3, on 106 patients. sealedenvelope software is used for randomization.

Settings and conduct

This study is a randomized clinical trial that will be performed on 3 to 7-day old neonates who are admitted due to jaundice after receiving the code of ethics for 8 months in 17 Shahrivar Hospital in Rasht. After selecting the infants according to the inclusion and exclusion criteria, written consent will be obtained from the parents and in the next step. Patients will be divided into 2 groups. Group A will receive oral Urso with phototherapy and group B will receive phototherapy alone (control). The drug is evaluated and visited by a Pediatric resident, and in case of complications, the drug will be discontinued. 1- Urso is administered orally at a dose of 10 mg/kg in divided doses every 12 hours and at the time of hospitalization. This dose will be dissolved in breast milk.

Participants/Inclusion and exclusion criteria

Inclusion criteria include the complete satisfaction of parents with the presence of children in the study, birth weight: 2500 to 4000 grams, exclusive breastfeeding, gestational age 38 to 41 weeks, age 3 to 7 days, total bilirubin 14 to 20 and less direct Of 2. Criteria for non-entry include ABO and RH incompatibility, G6PDd, direct hyperbilirubinemia, septicemia

Intervention groups

Urso at a dose of 10 mg/kg daily in infants receiving phototherapy during hospitalization

Main outcome variables

The primary outcome is bilirubin levels, which will be recorded within three to four days of hospitalization. The secondary outcome is the length of hospital stay - drug side effects and the timing of bilirubin testing.

General information

Reason for update

Acronym

IRCT registration information

IRCT registration number: **IRCT20210201050199N1**
Registration date: **2021-04-03, 1400/01/14**
Registration timing: **prospective**

Last update: **2021-04-03, 1400/01/14**
Update count: **0**

Registration date

2021-04-03, 1400/01/14

Registrant information

Name

manijeh Tabrizi

Name of organization / entity

Country

Iran (Islamic Republic of)

Phone

+98 13 3336 9002

Email address

drs.tabrizi@gmail.com

Recruitment status

Recruitment complete

Funding source

Expected recruitment start date

2021-04-19, 1400/01/30

Expected recruitment end date

2021-10-22, 1400/07/30

Actual recruitment start date

empty
Actual recruitment end date
empty
Trial completion date
empty

Scientific title
Therapeutic effect of oral ursodeoxycholic acid on indirect hyperbilirubinemia in full-term neonates undergoing phototherapy in hospital 17 Shahrivar - a randomized controlled clinical trial

Public title
Therapeutic effect of oral ursodeoxycholic acid on indirect hyperbilirubinemia in full-term neonates undergoing phototherapy in hospital 17 Shahrivar - a randomized controlled clinical trial
Purpose
Treatment
Inclusion/Exclusion criteria
Inclusion criteria:
Parents' complete satisfaction with the presence of children in the study Birth weight 2500 to 4000 grams Exclusive breastfeeding, Gestational age 38 to 41 weeks Age 3 to 7 days Total bilirubin 14 to 20 and direct bilirubin less than 2
Exclusion criteria:
ABO and RH incompatibility G6PD enzyme deficiency Direct hyperbilirubinemia Septicemia Diseases leading to hyperbilirubinemia (carpenter Kriegler syndrome, Gilbert syndrome, hyperthyroidism, hypothyroidism, liver disease) Preterm Neonate of mothers with gestational diabetes mellitus
Age
From **3 days** old to **7 days** old
Gender
Both

Phase
3
Groups that have been masked
No information
Sample size
Target sample size: **106**
Randomization (investigator's opinion)
Randomized
Randomization description
Randomization before the start of the program will be done by the software https://www.sealedenvelope.com with 53 people in each of the 2 groups (Urso and Photo and Control groups) in blocks of 4 and each of them inside A separate envelope is placed according to the list obtained from the software and is closed in it and will be given to the third person. If the patient visits and is eligible, the envelope is opened and treated according to the desired sequence without knowing the next treatment. . It should be noted that according to the grouping performed, patients will be divided into 2 groups based on random assignment done with the software. It is the responsibility of the pediatric resident to evaluate other variables and variables related to hyperbilirubinemia and to evaluate the side effects of the drug on a daily basis during hospitalization. Group A will receive oral Urso with phototherapy and group B will receive phototherapy alone (control). • 1, 4, 1, Group B • 1, 4, 2, Group B • 1, 4, 3, Group A • 1, 4, 4, Group A • 2, 4, 1, Group B
Blinding (investigator's opinion)
Not blinded
Blinding description
Placebo
Not used
Assignment
Parallel
Other design features

Secondary Ids

empty

Ethics committees

1

Ethics committee
Name of ethics committee
Ethics committee of the vice chancellor of Guilan University of Medical Sciences
Street address
Siadati
City
rasht
Province
Guilan
Postal code
4144444444
Approval date
2021-03-03, 1399/12/13
Ethics committee reference number
IR.GUMS.REC.1399.645

Health conditions studied

1

Description of health condition studied
Hyperbilirubinemia
ICD-10 code
E80.6
ICD-10 code description
Other disorders of bilirubin metabolism

Primary outcomes

1

Description
Total bilirubin
Timepoint
Every 24 hours
Method of measurement
Autoanalyzer device

Secondary outcomes

1

Description
Indirect bilirubin
Timepoint
Every 24 hours
Method of measurement
Autoanalyzer device

Intervention groups

1

Description
Intervention group: those who will receive oral Urso with phototherapy (administering Urso orally at a dose of 10 mg/kg in divided doses every 12 hours and at the time of hospitalization dissolved in breast milk)
Category
Treatment - Drugs

2

Description
Control group: those who will receive phototherapy alone
Category
Treatment - Drugs

Recruitment centers

1

Recruitment center
Name of recruitment center
17 Shahrivar Children's Hospital
Full name of responsible person
Manijeh Tabrizi
Street address
Siadati
City
Rasht
Province
Guilan
Postal code
4144444444
Phone
+98 13 3336 9002
Email
drs.tabrizi@gmail.com

Sponsors / Funding sources

1

Sponsor
Name of organization / entity
Rasht University of Medical Sciences
Full name of responsible person
Mohammadreza Naghipour
Street address
Siadati
City
Rasht
Province
Guilan
Postal code
4144444444
Phone
+98 13 3336 9002
Email
research@gums.ac.ir
Grant name
Grant code / Reference number
Is the source of funding the same sponsor organization/entity?
Yes
Title of funding source
Rasht University of Medical Sciences
Proportion provided by this source
100
Public or private sector
Public
Domestic or foreign origin
Domestic
Category of foreign source of funding
empty
Country of origin
Type of organization providing the funding
Academic

Person responsible for general inquiries

Contact
Name of organization / entity
Rasht University of Medical Sciences
Full name of responsible person
Manijeh Tabrizi
Position
Assistant Professor
Latest degree
Specialist
Other areas of specialty/work
Pediatrics
Street address
Siadati
City
Rasht
Province
Guilan
Postal code
4144444444
Phone
+98 13 3336 9002
Fax
Email
drs.tabrizi@gmail.com

Person responsible for scientific inquiries

Contact

Name of organization / entity
Rasht University of Medical Sciences
Full name of responsible person
Manijeh Tabrizi
Position
Assistant Professor
Latest degree
Specialist
Other areas of specialty/work
Pediatrics
Street address
Siadati
City
Rasht
Province
Guilan
Postal code
4144444444
Phone
+98 13 3336 9002
Fax
Email
drs.tabrizi@gmail.com

Person responsible for updating data

Contact
Name of organization / entity
Rasht University of Medical Sciences
Full name of responsible person
Manijeh Tabrizi
Position
Assistant Professor
Latest degree
Specialist
Other areas of specialty/work
Pediatrics
Street address
Siadati
City
Rasht
Province
Guilan
Postal code
4144444444
Phone
+98 13 3336 9002
Fax
Email
drs.tabrizi@gmail.com

Sharing plan

Deidentified Individual Participant Data Set (IPD)
No - There is not a plan to make this available
Justification/reason for indecision/not sharing IPD
Regarding the ethical issues and confidentiality
Study Protocol
No - There is not a plan to make this available
Statistical Analysis Plan
No - There is not a plan to make this available
Informed Consent Form
No - There is not a plan to make this available
Clinical Study Report
No - There is not a plan to make this available
Analytic Code
No - There is not a plan to make this available
Data Dictionary
No - There is not a plan to make this available
