# Supplementary material for: Evaluation of therapeutic effect of oral Ursodeoxycholic Acid on indirect hyperbilirubinemia in term neonates undergoing phototherapy: A randomized controlled clinical trial
Source: PLoS One. 2023 Dec 12;18(12):e0273516. doi: 10.1371/journal.pone.0273516 (PMC10715657; doi:10.1371/journal.pone.0273516)
Supplement: S3 File — (DOCX) [file pone.0273516.s005.docx]

We confirm that the clinical trial protocol we have included as Supplementary Information is the version that was submitted to and approved by your ethics committee before the trial began

You can find the Persian and English version as below

**پروتکل**

**هدف ازمطالعه**

مقایسه داروی اورسو خوراکی به همراه فو توتراپی با فوتوتراپی تنها در کاهش هیپربیلی روبینمی غیرمستقیم در نوزادان ترم تحت درمان فوتو تراپی بستری در بیمارستان 17شهریور

**طراحی**

کارآزمایی بالینی تصادفی دارای گروه کنترل

**نحوه و محل انجام مطالعه**

اين تحقيق يك مطالعه كارآزمايي باليني تصادفی شده است كه پس از اخذ کد اخلاق به مدت 6 ماه در بیمارستان 17 شهریور شهر رشت روی نوزادان ترم 3 تا 7 روزه که بدلیل زردی در بخش نوزادان بستری می شوند انجام شد. افراد واجد شرایط، طی تخصیص تصادفی (Random Allocation) به روش تصادفی بلوکه و یک به یک به دو گروه مداخله و کنترل تخصیص می یابند. برای تولید لیست تصادفی سازی افراد به گروه‌های مطالعه از سایت آنلاین https://www.sealedenvelope.com استفاده می شود که توسط شخص ثالث انجام و بر اساس روش بلوک‌های تصادفی و با درنظر گرفتن 27 بلوک‌ 4 تایی برای 108 بیمار در سایت تولید می شود.) با توجه به این که سایز بلوک 4 در نظر گرفته شده است ما می توانیم 6 توالی ممکن are AABB, ABAB, ABBA, BAAB, BABA, BBAA بر اساس خروجی سایت داشته باشیم و افراد را بر اساس آن تقسیم بندی کنیم (.

بعد از تولید لیست در سایت، به هر فرد یک کد اختصاصی تخصیص داده شده و در طول مطالعه فرد با این کد شناخته می شود. هیچ یک از افراد مشارکت‌کننده در اجرای مطالعه از لیست تصادفی‌سازی مطلع نیستند و نیز برای اعمال پنهان سازی فرآیند تصادفی سازی از پاکت های مهر و موم شده که به ترتیب شماره‌گذاری شده اند، استفاده و پاکت مربوط به هر فرد صرفاً بعد از تایید معیارهای واجد شرایط ورود به مطالعه برای او و امضای فرم رضایت‌نامه توسط فرد، باز می شود.

ثبت نام و توالی تخصیص تصادفی و اختصاص دادن مداخلات که نسبت به گروه مداخله و کنترل کور است توسط همکاران طرح انجام می‌پذیرد.

پس از انتخاب نوزادان با توجه به معیارهای ورود و خروج، رضایت نامه کتبی از والدین گرفته شد. بیماران در 2 گروه تقسیم بندی شد. گروه A دریافت کننده داروی اورسو خوراکی به همراه فوتو تراپی و گروهB دریافت کننده فوتوتراپی به تنهایی (کنترل) بود. اورسودئوکسی کولیک اسید خوراکی با دوز 10 ميلي گرم به ازاي هر كيلوگرم بصورت منقسم هر 12 ساعت و در زمان بستری تجویز شد که این دوز در شیر مادر حل شد.

**شرکت کنندگان/شرایط ورود و عدم ورود**

معیار ورود شامل: رضایت کامل والدین از حضورکودکان در مطالعه،وزن زمان تولد:2500 تا 4000 گرم،تغذیه ی انحصاری با شیر مادر،سن بارداری 38 تا 41 هفته،سن 3 تا 7 روز،بیلی روبین توتال 14 تا 20 و مستقیم کمتر از 1.5 می باشد. معیارهای عدم ورود شامل: ناسازگاری ABOو RH،کمبود آنزیم G6PD، کریگلر نجار، هیپربیلی روبینمی مستقیم،سپتیسمی و هیپوتیروئیدیسم و نوزاد مادر دیابتی.

**گروههای مداخله**

گروه مداخله علاوه بر فوتوتراپی، داروی اورسو ( کارخانه داروسازی امین) با دوز ۱۰ میلی گرم بر کیلوگرم روزانه در دو دز منقسم دریافت نموده و گروه کنترل تنها تحت فوتوتراپی قرار گرفتند.

**متغیرهای پیامد اصلی**

در این مطالعه تغییر در سطح بیلی روبین نسبت به سطح اولیه به عنوان پیامد اولیه و تفاوت در طول مدت بستری به عنوان پیامد ثانویه در نظر گرفته شد.

**کمیته اخلاق**

**نام کمیته اخلاق**

کمیته اخلاق دانشگاه علوم پزشکی گیلانٍ

**آدرس خیابان**

بیمارستان 17 شهریور.خیابان شهید سیادتی.خیابان نامجو

**شهر**

رشت

**استان**

گیلان

**کد پستی**

4144654839

**تاریخ تایید**

۱۳۹۹/۱۲/۱۳

**کد کمیته اخلاق**

IR.GUMS.REC.1399.645

**Protocol**

**Study aim**

Comparison of oral Ursodeoxycholic Acid (UDCA) with phototherapy and phototherapy alone in indirect hyperbilirubinemia reduction in term neonates undergoing phototherapy in hospital 17 Shahrivar

**Design**

Randomized controlled clinical trial

**Settings and conduct**

This study is a randomized clinical trial that will be performed on 3 to 7-day old neonates who were admitted due to jaundice after receiving the code of ethics for 6 months in 17 Shahrivar Hospital in Rasht. Eligible patients were randomly allocated by randomized blocking (or grouping). The following website “https://www.sealedenvelope.com” was used by a third party to produce the randomized list. Considering 27 blocks with the size of 4 and 6 possible variations (including AABB, ABAB, ABBA, BAAB, BABA, BBAA) were obtained. Then each patient received a specific code. Each code was placed in a separate sealed envelope. Finally an anonymous colleague assigned the eligible patients in the proper intervention and control groups after obtaining signed written informed consent. Parents/guardians were blinded to the groups enrolled. After selecting the infants according to the inclusion and exclusion criteria, informed written consent will be obtained from the parents and in the next step. Patients will be divided into 2 groups. Group A received UDCA with phototherapy and group B received phototherapy alone (control). UDCA was administered orally at a dose of 10 mg/kg in divided doses every 12 hours and at the time of hospitalization. This dose were dissolved in breast milk.

**Participants/Inclusion and exclusion criteria**

The inclusion criteria were 3 to 7 days of age, 2500 to 4000 grams birth weight, exclusive breastfeeding, 38 to 42 weeks of gestation, 14 - 20 mg/dL total serum bilirubin (TSB), and less than 1.5 mg/dL direct bilirubin. Neonates were excluded if there was ABO and Rh incompatibility, glucose 6 phosphate dehydrogenase deficiency (G6PDd), history of any neurological or chronic diseases, sepsis, diseases leading to hyperbilirubinemia (e.g., Crigler-Najjar and Gilbert's syndromes, hypothyroidism, and liver disease) and a history of maternal diabetes as well as parental dissatisfaction.

**Intervention groups**

The intervention group received 10 mg/kg/day oral UDCA divided into two doses plus phototherapy, which was dissolved in breast milk (Amin Pharmaceutical Company, Iran). The control group received phototherapy alone. The method of phototherapy was the same in both groups.

**Main outcome variables**

We considered the change of total serum bilirubin level from baseline as the primary outcome and the difference in length of hospitalization as the secondary outcome.

**Ethics committee**

**Name of ethics committee**

Ethics committee of Guilan University of Medical

Sciences

**Street address**

Namjou Ave,Shadid Siadati St,Rasht

**City**

Rasht

**Province**

Guilan

**Postal code**

4144654839

**Approval date**

2021-03-03

**Ethics committee reference number**

IR.GUMS.REC.1399.645
