# Supplementary material for: Evaluation of therapeutic effect of oral Ursodeoxycholic Acid on indirect hyperbilirubinemia in term neonates undergoing phototherapy: A randomized controlled clinical trial
Source: PLoS One. 2023 Dec 12;18(12):e0273516. doi: 10.1371/journal.pone.0273516 (PMC10715657; doi:10.1371/journal.pone.0273516)
Supplement: S4 File — (DOC) [file pone.0273516.s006.doc]

**
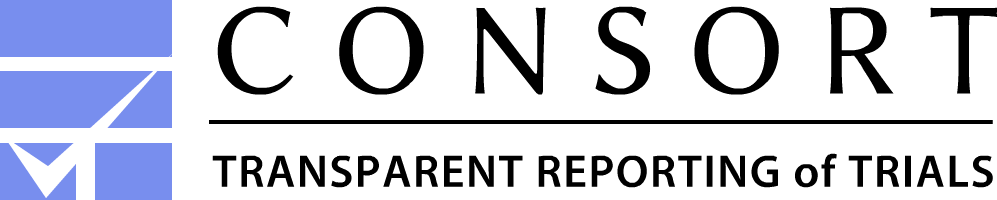
**

Assessed for eligibility

(n=198)

Randomized

(n=106)

UDCA + phototherapy

(n=53)

Phototherapy

(n=53)

Excluded (n=92)

Prematurity (n=50)

History of maternal diabetes (n=20)

Small for gestational age (n= 15)

Seizure (n= 4 )

Neonatal heart disease (n= 3 )

Excluded (n=10)

Lost to follow-up due to unwillingness

Excluded (n=4)

Lost to follow-up due to unwillingness

Analyzed

(n=43)

Analyzed

(n=49)

Fig 1) Flow chart of participants.
