# Supplementary material for: Evaluation of therapeutic effect of oral Ursodeoxycholic Acid on indirect hyperbilirubinemia in term neonates undergoing phototherapy: A randomized controlled clinical trial
Source: PLoS One. 2023 Dec 12;18(12):e0273516. doi: 10.1371/journal.pone.0273516 (PMC10715657; doi:10.1371/journal.pone.0273516)
Supplement: Attachment — Submitted filename: Dr Aghili response to comments.docx [file pone.0273516.s007.docx]

Dear editor in chief,

We appreciate your reviewers’ great comments and modified it based on your valuable suggestions.

When submitting your revision, we need you to address these additional requirements.

1. Please ensure that your manuscript meets PLOS ONE's style requirements, including those for file naming. The PLOS ONE style templates can be found at 
<https://journals.plos.org/plosone/s/file?id=wjVg/PLOSOne_formatting_sample_main_body.pdf> and <https://journals.plos.org/plosone/s/file?id=ba62/PLOSOne_formatting_sample_title_authors_affiliations.pdf>

We modified our article.

2. Please provide additional details regarding participant consent. In the ethics statement in the Methods and online submission information, please ensure that you have specified (1) whether consent was informed and (2) what type you obtained (for instance, written or verbal, and if verbal, how it was documented and witnessed). If your study included minors, state whether you obtained consent from parents or guardians. If the need for consent was waived by the ethics committee, please include this information.

If you are reporting a retrospective study of medical records or archived samples, please ensure that you have discussed whether all data were fully anonymized before you accessed them and/or whether the IRB or ethics committee waived the requirement for informed consent. If patients provided informed written consent to have data from their medical records used in research, please include this information.

We modified it and added the following statement:

This study was approved by the ethics committee of the Vice-Chancellor of Research at Guilan University of Medical Sciences (Number: IR.GUMS.REC.1399.645, Date: 2021-03-03). The clinical trial was registered in the Iranian registry of clinical trials (Number: IRCT20210201050199N1, Date: 2021-04-03). The authors confirm that all ongoing and related trials for this drug/intervention are registered. Written Informed consent letter was obtained from parents or guardians.

3. In your Data Availability statement, you have not specified where the minimal data set underlying the results described in your manuscript can be found. PLOS defines a study's minimal data set as the underlying data used to reach the conclusions drawn in the manuscript and any additional data required to replicate the reported study findings in their entirety. All PLOS journals require that the minimal data set be made fully available. For more information about our data policy, please see <http://journals.plos.org/plosone/s/data-availability>.

Upon re-submitting your revised manuscript, please upload your study’s minimal underlying data set as either Supporting Information files or to a stable, public repository and include the relevant URLs, DOIs, or accession numbers within your revised cover letter. For a list of acceptable repositories, please see <http://journals.plos.org/plosone/s/data-availability#loc-recommended-repositories>. Any potentially identifying patient information must be fully anonymized.

Important: If there are ethical or legal restrictions to sharing your data publicly, please explain these restrictions in detail. Please see our guidelines for more information on what we consider unacceptable restrictions to publicly sharing data: <http://journals.plos.org/plosone/s/data-availability#loc-unacceptable-data-access-restrictions>. Note that it is not acceptable for the authors to be the sole named individuals responsible for ensuring data access.

We will update your Data Availability statement to reflect the information you provide in your cover letter.

We modified it and uploaded the minimal underlying data set as an excel file.

4. Your ethics statement should only appear in the Methods section of your manuscript. If your ethics statement is written in any section besides the Methods, please delete it from any other section.

It was deleted and just noted the statement below as the second paragraph of the methods section.

This study was approved by the ethics committee of the Vice-Chancellor of Research at Guilan University of Medical Sciences (Number: IR.GUMS.REC.1399.645, Date: 2021-03-03). The clinical trial was registered in the Iranian registry of clinical trials (Number: IRCT20210201050199N1, Date: 2021-04-03). The authors confirm that all ongoing and related trials for this drug/intervention are registered. Informed written consent letter was obtained from parents or guardians.

5. Please include captions for your Supporting Information files at the end of your manuscript, and update any in-text citations to match accordingly. Please see our Supporting Information guidelines for more information: <http://journals.plos.org/plosone/s/supporting-information>.
[Note: HTML markup is below. Please do not edit.]

We modified it.

Reviewers' comments:

Reviewer #1: The authors conducted this study and aimed to investigate the effect of ursodeoxycholic acid on indirect hyperbilirubinemia in term neonates undergoing phototherapy. They concluded that UDCA plus phototherapy reduced total serum bilirubin and length of hospital stay with proper safety and efficacy. In general, the study design is adequate and well conducted with believable results. However, UDCA in the treatment of hyperbilirubinemia in neonates had been studied before as authors mentioned in the discussion. There are no novel findings in the present study. Besides, authors should discuss more regarding the discrepancies between the present and previous studies.

We appreciate your comment. You are completely right, but as we mentioned based on the controversial results regarding the use of UDCA on hyperbilirubinemia and the shortage of evidence about its side effects, the aim of this study was to evaluate the therapeutic effect of oral UDCA plus phototherapy in the management of neonatal hyperbilirubinemia and to identify any side effects of this treatment

Other comments list below.

1. In conclusions of main text and Abstract. “Administering UCDA plus phototherapy reduced TSB….” It should be “UDCA”, not “UCDA”.

We appreciate your precise suggestion and we corrected it.

2. Is the effect of UDCA different in different subgroup of neonates like male/female. Authors should perform subgroup analysis to understand whether similar effect of UDCA in different subgroups.

We appreciate your comment and consult with our statistician. She declared that due to no significant difference between the two groups regarding age, weight, and sex, there was no need to control the effect of variables by subgroup and regression analyses.

3. A table of baseline demographics should be added in the main text.

We appreciate your comment and added the following Table 1.

Table 1: Baseline demographic characteristics in the intervention and control groups.

| **Variables** | **Intervention group (n=53)** | **Control group**  **(n=53)** | **P Value** |
| --- | --- | --- | --- |
| age(yr)  mean ± SD | 5.10 ± 1.25 | 5.18 ± 2.26 | 0.464 |
| weight(gr)  mean ± SD | 3263.02 ± 364.81 | 3207.74 ± 400.17 | 0.459 |
| Sex (male)  N(%) | 28 (52.8 %) | 29 (54.7%) | 0.846 |

4. Is the total serum bilirubin level significantly different between intervention and control groups at 12, 24, and 48 hours? Authors may also add a figure drawing the decline of bilirubin levels of all/intervention/control groups from baseline to discharge.

We added the following figure in our main text.

|  | Intervention group (n=49) | Control group(n=43) | all groups |
| --- | --- | --- | --- |
| Bilirubin at the discharge (mg/dl) | 7.73 | 8.61 | 8.14 |
| Bilirubin at 48 hours after hospitalization (mg/dl) | 10.09 | 11.83 | 10.9 |
| Bilirubin at 24 hours after hospitalization (mg/dl) | 13.73 | 14.24 | 13.97 |
| Bilirubin at 12 hours after hospitalization (mg/dl) | 17.68 | 17.3 | 17.5 |
| Bilirubin at the admission (mg/dl) | 17.678 | 17.3 | 17.5 |

Figure 2: The trend of bilirubin levels of all, intervention, and control groups from baseline to discharge.

5. The results showed combined UDCA therapy had significantly lower TSB level at discharge and shorter duration of hospitalization. Authors should further adjust UDCA therapy with other associated factors to confirm the independent association of UDCA therapy.

We appreciate your comment and consult with our statistician. She declared that due to no significant difference between the two groups regarding age, weight, and sex, there was no need to control the effect of variables by subgroup and regression analyses.

Reviewer #2: This a study evaluating the therapeutic effect of oral Ursodeoxycholic Acid plus phototherapy in the management of neonatal hyperbilirubinemia.

1. It would be good if the authors expanded on the content in the introduction section. To include rationale for the proposed RCT. Also it would be good to include more studies that have also looked at similar effects and why this study is so important.

We appreciate your comment. We added the following explanation in the introduction section.

It is noteworthy that pediatricians have to be assured about the safety profile of drugs used for neonates. Although some previous studies mentioned the positive effect of administering UDCA on neonatal hyperbilirubinemia (5-10), yet there is no consensus on its routine use. Therefore, it is not prescribed as an adjunct treatment in neonatal wards and further studies are needed. Based on the controversial results regarding the use of UDCA on hyperbilirubinemia and the shortage of evidence about its side effects, the aim of this study was to evaluate the therapeutic effect of oral UDCA plus phototherapy in the management of neonatal hyperbilirubinemia and to identify any side effects of this treatment. We hypothesized that administering UDCA plus phototherapy may have a significant effect on the change of TSB level from baseline as the primary outcome and the difference in length of hospitalization as the secondary outcome with no considerable side effect.

2. The randomisation section is very brief, can the authors elaborate on who was blinded and also more details with regards to how allocation concealment was ensured. Its not clear from the sentence “According to the list obtained from the software and given a block size of 4, each case was placed in a separate envelop and its lid was closed and given to a third party”

We appreciate your comment. We added the following paragraph.

Eligible patients were randomly allocated by randomized blocking (or grouping). The following website “https://www.sealedenvelope.com” was used by a third party to produce the randomized list. Considering 27 blocks with the size of 4 and 6 possible variations (including AABB, ABAB, ABBA, BAAB, BABA, BBAA) were obtained. Then each patient received a specific code. Each code was placed in a separate sealed envelope. Finally an anonymous colleague assigned the eligible patients in the proper intervention and control groups after obtaining signed written informed consent.

3. Additionally can the authors explicitly say who was blinded. From reading line 87, it implies Eligible patients were blinded, presumably do you mean parents/guardians since these were neonates who would not even assimilate what is going on.

You are completely right. Parents/guardians were blinded to the groups enrolled.

4. Can the authors give more details on the sample size, as it does not contain the effect size of the outcome and so impossible to replicate.

We appreciate your comment.

Sample size was indicated based on Shahramian et al. (6). With the confidence interval of 95% and 80% test power, the sample size was equal to 48 neonates in each group. Considering the drop rate of 10% during the study, 53 neonates in each group were required.

n_2_=48

10% drop rate=5

N= 53 in each group

Also, we checked our study power and you can find it below:

5. The methods needs to include details and definitions of outcomes. i.e saying “we considered TSB level as the primary outcome” is not enough as you need to define what exactly you looking for. For example is it change of TSB from baseline. Same comment for secondary outcome, i.e. length of hospitalization.

We appreciate your great recommendation. We considered the change in TSB level from baseline as the primary outcome and the difference in length of hospitalization as the secondary outcome.

6. A minor comment, when the authors mention data collected. For example makes it clear to say what was collected at baseline and what was collected post randomisation.

We appreciate your great recommendation. Data were gathered by a checklist including baseline and post randomization variables. Baseline characteristics were age, sex, gestational age, and baseline blinrubin level. Also, bilirubin level at 12, 24, and 48 hours after admission and side effects including nausea, vomiting, constipation, bloating, and skin rash were assessed as post randomization variables in the two groups.

7. The last paragraph in the introduction should make clear the primary (i.e effect of xxxx on total serum bilirubin) and secondary objectives (length of hospitalization) and safety objectives.

We appreciate your comment. In response to your great recommendation, we added the following statement. We hypothesized that administering UDCA plus phototherapy may have a significant effect on the change of TSB level from baseline as the primary outcome and the difference in length of hospitalization as the secondary outcome with no considerable side effect.

8. The statistical analysis is very vague on the analysis of the specific outcomes.

We appreciate your comment. In order to clarify the results, we added all different statistical tests in the tables. Because as we all know, adding these parts can indicate the types of data and their distribution.

9. As this is an RCT, there should be no formal comparisons made between baseline characteristics as any differences observed will be by chance.

We appreciate your comment. We used this comparison to be assured about the similarity of variables distribution in the two groups. As we all know, in case of small sample size, randomization and randomized allocation may not similarize the variables’ distribution in the two groups. Therefore, we always assess this issue. If there was different distribution, the investigator must use statistical analysis to control its effect while assessing the relation between dependent and independent variables.

10. An observation is that the attached protocol is a summary and should be the full protocol attached. As the study is complete now, therefore would serve useful information to accompany the manuscript.

We modified it in the attached file.

Reviewer #3: The authors aimed to evaluate the therapeutic effect of oral ursodeoxycholic acid (UDCA) on indirect hyperbilirubinemia in term neonates undergoing phototherapy in a randomized controlled clinical trial. They found that at the time of discharge, total serum bilirubin (TSB) level in the combined treatment compared to the latter group was significantly reduced (7.74± 1.39 vs. 8.67±1.35). In addition, the duration of hospitalization was considerably shorter in the intervention compared to the control group. They concluded that combined UCDA plus phototherapy can be an appropriate treatment modality in neonatal hyperbilirubinemia. The comments were as followed.

1. There were no any creative viewpoints in this study compared to previous published data with similar study design and results.

We appreciate your comment. You are completely right, we mentioned the following paragraph.

It is noteworthy that pediatricians have to be assured about the safety profile of drugs used for neonates. Although some previous studies mentioned the positive effect of administering UDCA on neonatal hyperbilirubinemia (5-10), yet there is no consensus on its routine use. Therefore, it is not prescribed as an adjunct treatment in neonatal wards and further studies are needed. Based on the controversial results regarding the use of UDCA on hyperbilirubinemia and the shortage of evidence about its side effects, the aim of this study was to evaluate the therapeutic effect of oral UDCA plus phototherapy in the management of neonatal hyperbilirubinemia and to identify any side effects of this treatment. We hypothesized that administering UDCA plus phototherapy may have a significant effect on the change of TSB level from baseline as the primary outcome and the difference in length of hospitalization as the secondary outcome with no considerable side effect.

2. All participants who are randomized in an intention-to-treat analysis should be included in the statistical analysis.

We appreciate your comment. As you know, one of the conditions for using Friedman analysis is the equal number of participants in the two groups. Therefore, we cannot assess all participants in this section. We modified our statement as below.

We intended to investigate the difference in bilirubin levels in these three time periods, the neonates studied at this stage were 49 in the intervention and 43 in the control group. Therefore, 14 participants were excluded due to loss to follow-up.

3. Table 2. The analysis of bilirubin level in 12, 24, and 48 hours after hospitalization should be compared between intervention and control group.

We appreciate your comment. As we added Table 1 in the main text for demographic characteristics, we mentioned these phrases. The intragroup analysis indicated that TSB in both groups had significant decrease during 12, 24, and 48 hours after hospitalization (P <0.001). Although there was no significant difference between the two groups at 12 and 48 hours after hospitalization (P>0.05), significant difference was noticed at 48 hours after hospitalization (P=0.009) (Table 3).

Table 3: Inter and intra group analyses of bilirubin level in 12, 24, and 48 hours after hospitalization.

| **P- value*** | Bilirubin at 48 hours after hospitalization (mg/dl)  mean ± SD | Bilirubin at 24 hours after hospitalization (mg/dl)  mean ± SD | Bilirubin at 12 hours after hospitalization (mg/dl)  mean ± SD | **Groups** |
| --- | --- | --- | --- | --- |
| <0.001 | 10.09±2.48 | 13.73±2.6 | 17.68±1.85 | Intervention group (n=49) |
| <0.001 | 11.83±1.33 | 14.24±2.16 | 17.30±1.83 | Control group  (n=43) |
|  | 0.009 | 0.271 | 0.351 | P- value** |

*Friedman test

*** Mann-Whitney U test

4. How did the authors explain no differences in TSB level at 12, 24 and 48 hours, but significantly reduction at the time of discharge between intervention and control groups?

We appreciate your comment. As we all know, phototherapy is the main treatment modality for hyperbilirubinemia. In this study, we added UDCA as an adjunct to phototherapy. It is logical to expect decreased bilirubin level in both groups. When we compared groups at 48 hours, we found lower TSB level in the intervention than the control. Therefore, the lower bilirubin level in the intervention group can decrease the length of hospital stay.
